# Editorial: Women in fungal pathogenesis 2021

**DOI:** 10.3389/fcimb.2022.1068446

**Published:** 2022-11-04

**Authors:** Angie Gelli, Clarissa J. Nobile, Eva Pericolini, Melanie Wellington

**Affiliations:** ^1^ Department of Pharmacology, School of Medicine, University of California, Davis, Davis, CA, United States; ^2^ Department of Molecular and Cell Biology, School of Natural Sciences, University of California, Merced, Merced, CA, United States; ^3^ Health Sciences Research Institute, University of California, Merced, Merced, CA, United States; ^4^ Department of Surgical, Medical, Dental and Morphological Sciences with Interest in Transplant, Oncological and Regenerative Medicine, University of Modena and Reggio Emilia, Modena, Italy; ^5^ Department of Pediatrics, Carver College of Medicine, University of Iowa, Iowa City, IA, United States

**Keywords:** fungal pathogenesis, mycology, dermatophytosis, fungal dysbiosis, antifungal susceptibility testing, women scientists in mycology, candida and candidiasis

## Introduction

This Research Topic consists of seven articles that have been viewed a total of 11,874 times as of October 2022. The aim of this special topic was to assemble a range of topics that reflected the work of women studying fungal pathogenesis. While gender parity exists when examining the number of women studying mycology (51.5%) and those listed as first authors (49.1%), only 1/3 of those were corresponding authors (36.4% and 32.7%, respectively) (Krasevec, 2022). This is consistent with the gender gaps that persist in the STEM fields at senior level positions (Holman et al., 2018). Recent estimates suggest that requests for article submission by scientific journals favor men over women two to one (Holman et al., 2018). This same study found a negative correlation between journal impact factor and the number of women authors (Holman et al., 2018). The goal of this Research Topic was to underscore the obstacles that remain in place for women researchers, promote awareness within the scientific community of the contributions female researchers have made to the field, and promote the interest of young women scientists in this topic.

## Mycobiome dysbiosis in alcoholism

The article by Day and Kumamoto [*Gut microbiome dysbiosis in alcoholism: Consequences for health and recovery*], focused in part on the differences in the fungal microbiome of the gastrointestinal tract in individuals who abuse alcohol versus those who do not. While most studies have focused on the bacterial microbiome of individuals with alcohol use disorder (AUD), very few have examined the mycobiome and its health effects on those with AUD, on the progression of alcohol-associated liver disease (ALD) and on its potential impact on the gut-brain axis. This enlightening review suggests that although large gaps in understanding the role of the mycobiome in AUD persist, recent studies suggest that alcohol abuse leads to fungal dysbiosis in the gut. The significant changes noted in the AUD mycobiome include an increase in the abundance of *Candida* and *Pichia* species, suggesting that those with AUD may be at greater risk of developing infections from these fungal pathobionts (Hartmann et al., 2021). While the molecular mechanisms mediating fungal dysbiosis have not been resolved, the authors propose that disruption of bacterial microbiome homeostasis in AUD may promote fungal blooms in the GI tract of those with AUD.

## *Candida* biology and candidiasis: Interactions between fungi and the host

Studies on the biology and pathogenesis of *Candida* dominated this Research Topic. *Candida* species run the spectrum from benign common fungal commensals in the mycobiome to opportunistic pathogens that can cause potentially life-threatening systemic infections. One of the most studied transcriptional regulators in *Candida* biology is Efg1. Beautifully reviewed by Glazier [*EFG1, everyone’s favorite gene in Candida albicans: A comprehensive literature review*], this thorough and wide-ranging review provides an in-depth discussion of the roles of *EFG1* from filamentation, morphogenesis, and biofilm regulation to its opposing functions in commensalism and invasive infection. Despite the intense study of *EFG1* and the 25 years since *EFG1* was first characterized in *Candida albicans*, the author raises several intriguing and unresolved questions such as what are the processes that regulate post-transcriptional expression of *EFG1* and how do these impact *EFG1* activity in the host?

The interplay between *Candida* and the host response during the course of oral candidiasis was examined in a brief research article by Beute et al. [*The IL-20RB receptor and the IL-20 signaling pathway in regulating host defense in oral mucosal candidiasis*]. In this exciting study, the authors found that a newly recognized keratinocyte pathway, the IL-20 cytokine signaling pathway, plays a role in regulating host defense in oral mucosal candidiasis.

In related studies, an excellent research article by Harriett et al. [*Efficacy of Candida dubliniensis and fungal beta-glucans in inducing trained innate immune protection against inducers of sepsis*] found that immunization with *Candida dubliniensis* or fungal cell components can induce Gr-1+ cell-mediated trained innate immune protection against sepsis and that beta-glucans may be sufficient for protection against lethal polymicrobial sepsis.

Fungal and bacterial co-infections can be particularly damaging to the host. In an excellent research article by Niemiec et al. [*Augmented enterocyte damage during Candida albicans and Proteus mirabilis coinfection.*] fungal-bacterial interactions were investigated by co-infecting enterocytes with *Candida albicans* and *Proteus mirabilis*, a gram-negative bacterium. The authors found that the virulence of *Proteus* and the resulting host cell damage may have been triggered by *Candida*-mediated glucose consumption and farnesol production. This exciting study emphasizes the notion that cross-kingdom interactions can potentially alter microbial virulence in host microenvironments.

## Dermatophytosis and antifungal susceptibility testing

Unlike the systemic fungal pathogens, dermatophytes are fungi that cause superficial infections of keratin-rich tissue such as skin, nails, and hair. While dermatophytes almost never cause life-threatening infections, dermatophytosis causes significant morbidity and distress to those afflicted. For individuals with diabetes, skin infections caused by dermatophytes are especially troubling. In this exciting study by Nabeela et al. [*Antifungal activity of alexidine dihydrochloride in a novel diabetic mouse model of dermatophytosis.*], a model of dermatophytosis in diabetic mice was developed. Using this model, mice with fungal lesions that were treated with alexidine dihydrochloride, a drug with newly discovered antifungal properties, displayed similar outcomes to mice treated with terbinafine, a well-known antifungal drug used widely by clinicians. This animal model will be particularly useful in assessing the potency of new candidate antifungal drugs in the treatment of dermatophytosis.

For clinicians to successfully treat fungal infections, trends in antifungal resistance must be monitored. Antifungal susceptibility testing of fungal pathogens is a common practice. In a research article by Pellaton et al. [*How yest antifungal resistance gene analysis is essential to validate antifungal susceptibility testing systems.*], the authors compared two commercial microdilution antifungal susceptibility testing methods (YO and MNV) to detect antifungal resistance of fungal strains with point mutations in antifungal resistant genes. The authors report that both methods are highly useful but individual advantages and limitations should be considered before implementation and during analysis. The authors also caution that conclusive analyses are difficult without the use of antifungal gene sequence data as a gold standard.

## Conclusion

This collection of studies highlights some of the outstanding work performed by women scientists committed to analyzing the interplay between fungi and the host, discovering new mechanisms of pathogenesis and new potential therapeutic targets. Achieving gender parity at the most senior level of scientific research will require more action. This Research Topic underscoring women’s contributions to the field of mycology is a step forward.

## Author contributions

AG wrote the draft, CN, EP and MW contributed to the final editorial. All authors contributed to the article and approved the submitted version.

## Conflict of interest

CN is a cofounder of BioSynesis, Inc., a company developing diagnostics and therapeutics for biofilm infections.

All other authors declare that the research was conducted in the absence of any commercial or financial relationships that could be construed as potential conflict of interest.

## Publisher’s note

All claims expressed in this article are solely those of the authors and do not necessarily represent those of their affiliated organizations, or those of the publisher, the editors and the reviewers. Any product that may be evaluated in this article, or claim that may be made by its manufacturer, is not guaranteed or endorsed by the publisher.
